# Mechanical fatigue in microtubules

**DOI:** 10.1038/s41598-024-76409-7

**Published:** 2024-11-01

**Authors:** Syeda Rubaiya Nasrin, Neda M. Bassir Kazeruni, Juan B. Rodriguez, Stanislav Tsitkov, Akira Kakugo, Henry Hess

**Affiliations:** 1 Division of Physics and Astronomy, Graduate School of Science, Kyoto University, Kitashirakawa-Oiwake-Cho, Sakyo-ku, Kyoto, 606-8502 Japan; 2 Department of Biomedical Engineering, Columbia University, 1210 Amsterdam Avenue, New York, NY 10027 USA; 3 Department of Biological Engineering, Massachusetts Institute of Technology, Cambridge, MA USA

**Keywords:** Fatigue failure, Fatigue strength exponent, Microtubule, Tubulin, Mechanobiology, Motor protein tracks, Biomedical engineering

## Abstract

Mechanical failure of biological nanostructures due to sustained force application has been studied in great detail. In contrast, fatigue failure arising from repeated application of subcritical stresses has received little attention despite its prominent role in engineering and potentially biology. Here, paclitaxel-stabilized microtubules are up to 256 times bent into sinusoidal shapes of varying wavelength and the frequency of breaking events are observed. These experiments allow the calculation of fatigue life parameters for microtubules. Repeated buckling due to 12.5% compression–equal to the compression level experienced by microtubules in contracting cardiomyocytes – results in failure after in average 5 million cycles, whereas at 20.0% compression failure occurs after in average one thousand cycles. The fatigue strength (Basquin) exponent B is estimated as − 0.054±0.009.

**Supplementary Information:**

The online version contains supplementary material available at 10.1038/s41598-024-76409-7.

## Introduction

Nanomechanical studies of biological structures have delivered important biophysical insights which advanced our understanding of cell biology^1^. In particular, the characterization of the mechanical properties of cytoskeletal filaments, such as actin filaments and microtubules, has attracted great interest due to their prominent roles in cell mechanics^2–4^, but also increasingly as scaffolds and motile agents for hybrid nanodevices^5–8^. From the initial measurements of their rigidity^9–11^, over indentation^12,13^ and breaking^6,14^ experiments to the recent discovery of their self-healing capabilities^15^, cytoskeletal filaments have revealed a surprising complexity in their mechanical properties. For example, the breaking rate of microtubules increases exponentially with increasing curvature^16^, a fact which can be understood based on mechanochemical principles^17^ and which affects the organization of the microtubule cytoskeleton^18^. However, microtubules in vitro and in vivo do not only experience constant or increasing loads which cause breaking, but also the repeated application of subcritical stresses.

**Fig. 1:**
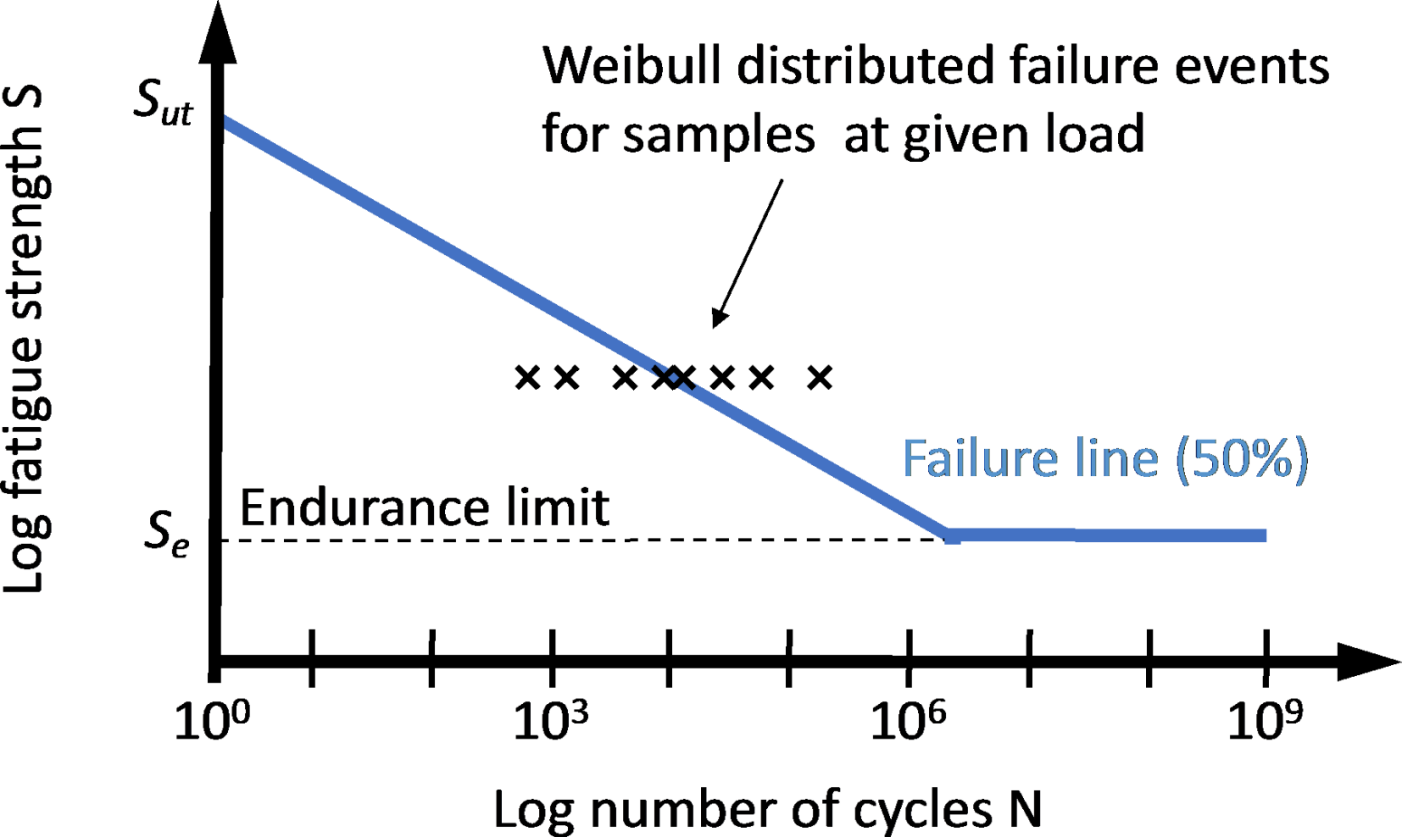
S-N diagrams are used in engineering to describe the fatigue behavior of mechanical parts (axles, beams, etc.). Samples of these parts are exposed to cyclic loading at a defined stress level until failure occurs. The number of cycles to failure varies from part to part, and the failure line indicates the number of cycles after which 50% of the samples have failed for a given load. At the ultimate tensile strength, S_ut_, the part fails during the first loading cycle. Some materials (e.g. steel) exhibit an endurance limit, S_e_, below which fatigue failure is not observed. Other materials experience fatigue failure eventually under any load. The fatigue strength (Basquin) exponent is the slope of the failure line in the log-log plot with typical values ranging from − 0.05 to − 0.12 for metals.

The repeated application of subcritical stresses leads to materials fatigue in macroscopic systems^19^, where each stress cycle can cause the incremental growth of cracks leading ultimately to fracture of the part and failure of the system. The fatigue properties of a material are typically characterized using an S-N diagram, where the average number of stress cycles until failure is related to the applied stress (Fig. 1). While application of the ultimate stress leads to failure in a single stress cycle, decreasing the stress below the ultimate stress typically leads to a large increase in the number of cycles before failure is observed^19^. Some materials exhibit an endurance limit, that is, a stress level below which failure is not observable anymore.

Here we aim to experimentally measure the S-N diagram for paclitaxel-stabilized, fluorescently labeled microtubules by repeatedly buckling straight microtubules into sinusoidal shapes. This is accomplished by tethering microtubules via kinesin motor proteins to a flexible polydimethylsiloxane (PDMS) substrate, which is stretched and relaxed by external actuators^20^. Each relaxation-stretch cycle of the pre-stretched PDMS substrate results in a buckling-straightening cycle of the microtubules immobilized on it. The integrity of the microtubules after the completion of up to 256 cycles was observed using fluorescence microscopy.

**Fig. 2:**
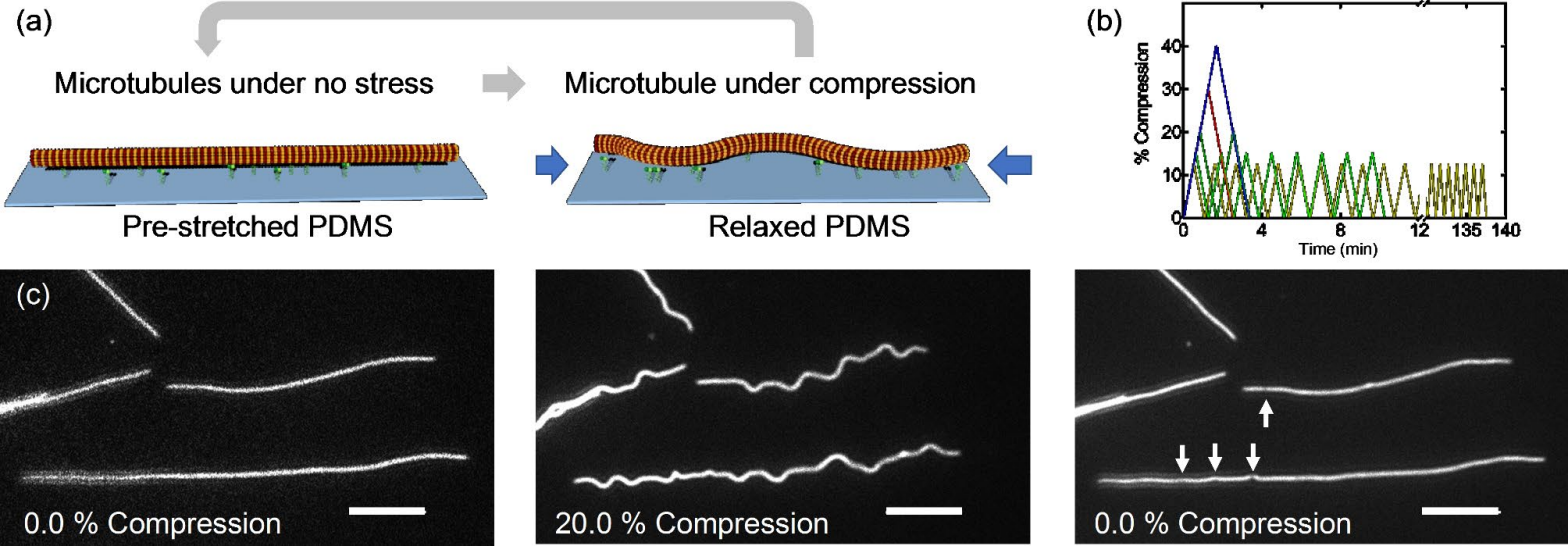
Sketch of the setup. (**a**) Fluorescently labeled microtubules were immobilized on kinesin coated PDMS sheet pre-stretched by a defined amount. The sheet is relaxed by a certain fraction of its stretched length causing the buckling of the microtubules via forces exerted by the kinesin cross-linkers to the surface. The PDMS sheet is then stretched again to return the microtubules to their linear configuration and complete the loading cycle. (**b**) The compression level (negative strain) as a function of time. The strain rate is 0.004 s^−1^ in all experiments, and 1 s passes between motion reversals. (**c**) Fluorescence microscopy images of the microtubules at different stages of the experiment for 20% compression. Arrows denote breaks identified by visual inspection. Scale bar: 10 μm.

Repeated bending of microtubules into reproducible sinusoidal shapes was accomplished using a “micro-stretcher”^20,21^ which actuates a two-dimensional elastic film of PDMS used as substrate to immobilize the microtubules via kinesin-1 motor proteins bound to the substrate (pre-stretched to 100% strain). The ATTO 488-labeled microtubules are largely aligned with the axis of stretching by shear flow during their initial interaction with the kinesin-covered PDMS surface. Continuous purging of the experimental chamber with humid N_2_ minimized evaporation and photodamage over several hours of observation with fluorescence microscopy^22^. The buffer solution did not contain ATP or ADP in order to attach the microtubules to the kinesin in the strongly bound rigor state (Fig. 2a).

## Results

The experiment proceeded by repeatedly relaxing and stretching the pre-stretched PDMS at a strain rate of 0.004 s^−1^ (with 1 s intervals between each motion), which causes microtubules to buckle into sinusoidal waves and straighten again (Fig. 2b,c). Variations in the kinesin density on the surface lead to variations in buckling amplitude along each microtubule. By analyzing small segments of each microtubule and determining the curvature of each segment individually, we can investigate the breaking rate for a specific curvature^16^. The sinusoidal shape of the buckled microtubule prevents the build-up of internal stresses between protofilaments described by Pampaloni et al. for bending microtubules with increasing length^11^, so that the effective persistence length is expected to be small and on the order of 0.2 mm as for very short microtubules. The extent of buckling is controlled by the degree of compression relative to the initial pre-stretched state of the PDMS substrate^23^. Since our current experimental setup allows compressive strain of up to 40%, we chose compression levels of 10.0%, 12.5%, 20.0%, 30.0% and 40.0%, requiring cycle completion times between 52 s (10.0%) and 202 s (40.0%).

Images of the microtubules were captured after specific numbers of stretch cycles (1, 2, 4, 8, 16, 32, 64, 256; Fig. 2c). Breakage or damage of a microtubule was visually identified by a non-uniformity in the fluorescence signal of a microtubule (Fig. 2c). In some cases, the breaks on the microtubules that were not observable in the buckled state became visible in the stretched state.

**Fig. 3:**
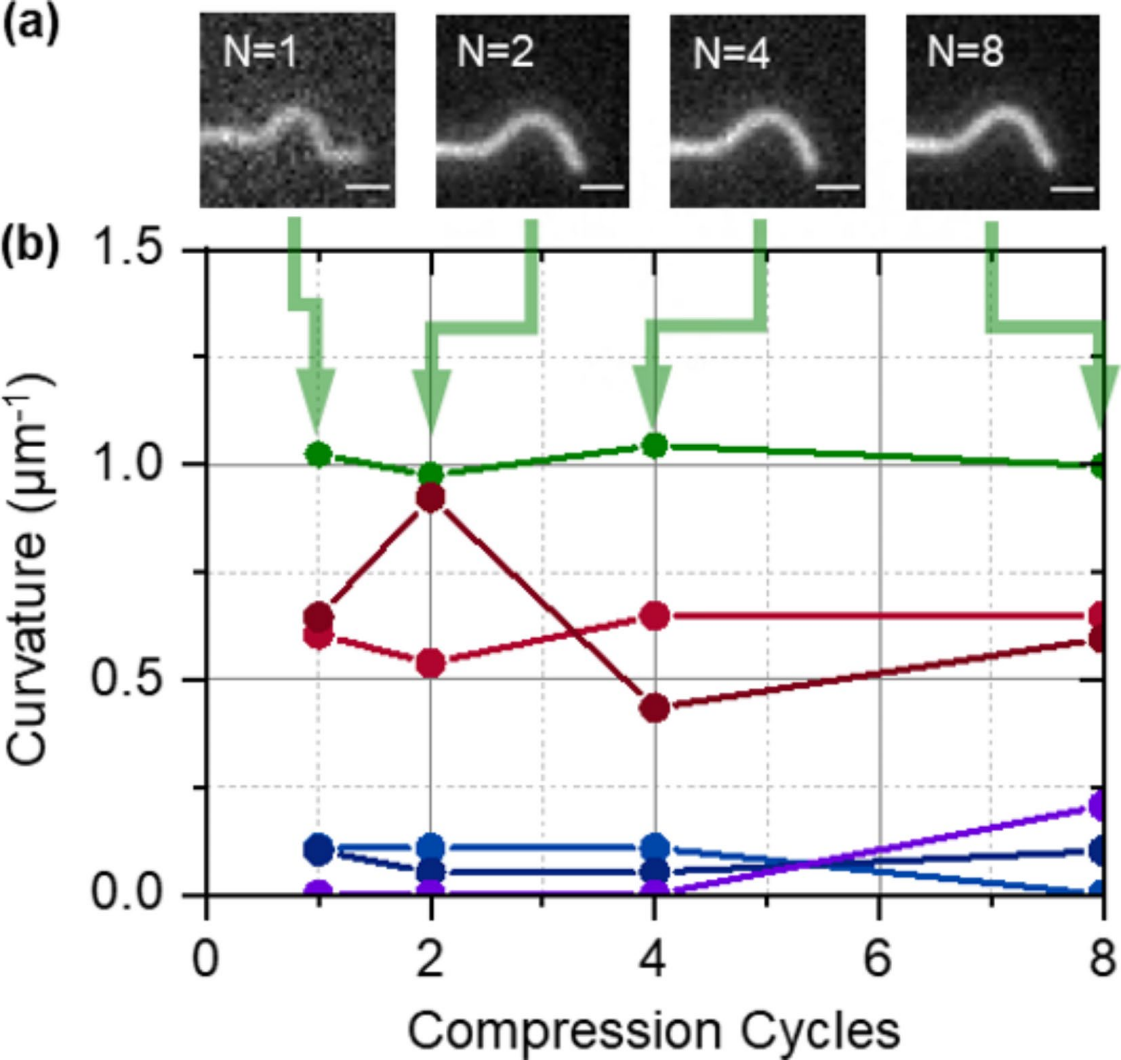
The curvature of microtubule segments remains constant over many compression cycles. (**a**) Images of a high curvature microtubule segment after 1, 2, 4, and 8 cycles of 20% compression and relaxation (scale bar: 1 μm). (**b**) Curvature of 6 individual microtubule segments after 1, 2, 4, and 8 compression cycles. The microtubule segment shown in (**a**) corresponds to the highest curvature segment plotted in (**b**).

At the highest compression level of 40.0%, a single compression already creates breakage along the microtubules, which becomes more clearly visible after the microtubules are returned to the stretched state (Supplementary Fig. S1). At lower compression levels, a single cycle was insufficient to break the microtubules (Supplementary Fig. S1). At 10.0%, even 256 cycles did not disrupt the microtubules. We therefore analyzed in detail 1 to 8 cycles for a compression level of 20.0% and 1 to 256 cycles for a compression level of 12.5%. All microtubules were identified and divided into 0.5 μm long segments for which the curvature after compression was determined. The segment length was chosen because it is optimal for determining the curvature^16^. Specific segments repeatedly experienced the same level of curvature (Fig. 3). Breaking events were identified by close examination by eye and associated with specific segments. The microtubule segments at risk and the microtubule segments experiencing a break were grouped into curvature bins (0–0.5, 0.5–1, …, 2.5–3 μm^−1^) as shown in Table 1, because the curvature determines the local breaking rate^16^.

**Fig. 4:**
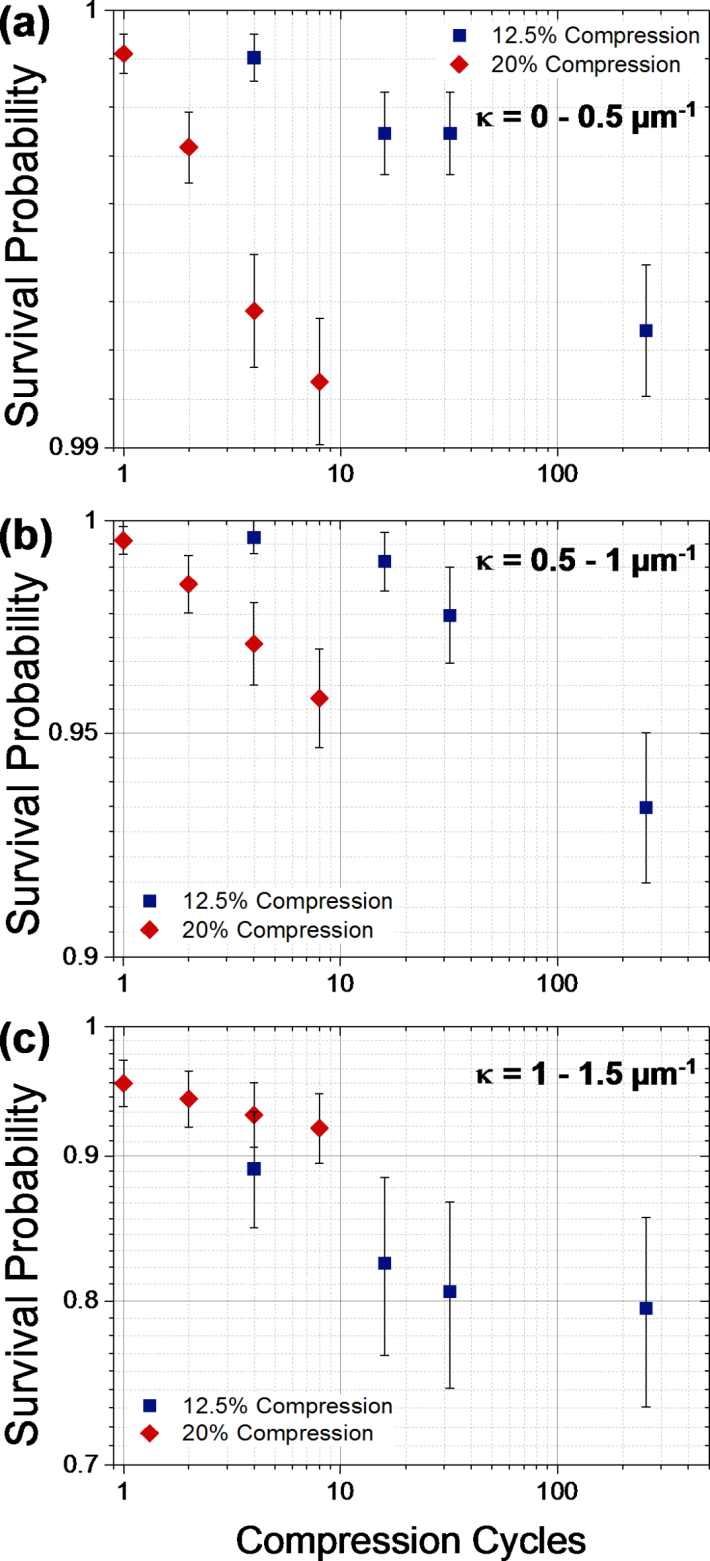
The survival probability of microtubule segments as a function of the number of strain cycles for the two compression ratios (12.5% and 20.0%) and three curvature bins with curvatures between (**a**) 0.0 and 0.5 μm^−1^, (**b**) 0.5 and 1.0 μm^−1^, (**c**) 1.0 and 1.5 μm^−1^ for which more than one break was observed. Error bars represent standard error.

The number of microtubule segments at risk and the number of new microtubule segments breaking allows the calculation of the logarithm of the complement of the breaking probabilities (the survival probability) as a function of the logarithm of the cycle number (Fig. 4). The breaking probability exhibits the expected strong dependence on the curvature and increases roughly linearly with the logarithm of the cycle number. For example, for the 20.0% compression level and after 8 cycles, the breaking probability increases from 0.009 over 0.04 to 0.08 for the 0.0–0.5, 0.5–1.0 and 1.0–1.5 μm^−1^ curvature bins, respectively. However, the survival curves for the 12.5% and 20.0% compression levels do not overlap, as one would expect if curvature and cycle number are the only determinants of survival. The absence of a consistent trend for the different curvature conditions points towards a significant stochastic variability between experiments.

**Fig. 5:**
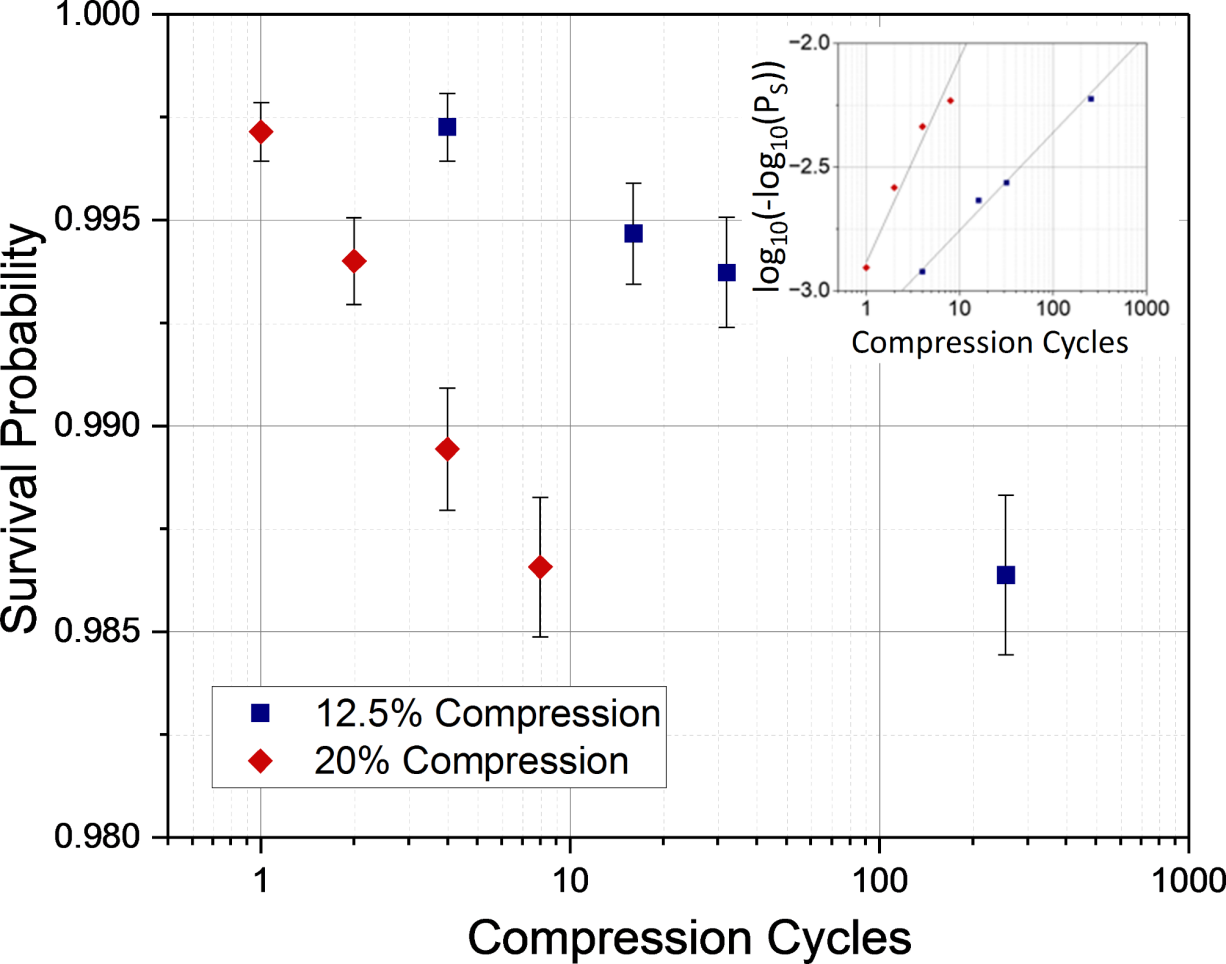
The survival probability of microtubule segments as a function of the number of strain cycles for the two compression ratios (12.5% and 20.0%) independent of curvature. The inset shows the linear fit to a Weibull distribution.

To reduce stochastic effects, we therefore pool the different curvature bins and calculate a survival curve for each compression level (Fig. 5) and model it as a Weibull distribution^24,25^:

1 $$\:{P}_{S}\left(N\right)={e}^{-{\left(N/b\right)}^{a}}$$

For our experiments, the shape and scale coefficients a and b were determined as 0.39 ± 0.03 and 10^7^ for 12.5% compression, and 0.83 ± 0.11 and 10^3^ for 20.0% compression, respectively. From these fits the number of cycles N_50_ at which 50% of the segments are broken can be extrapolated to be 5 × 10^6^ for 12.5% compression and 10^3^ for 20.0% compression.

**Table 1:** Number of microtubule segments exposed to different curvatures and the associated number of breaks.

Number of cycles	κ = 0.0–0.5 [µm^−1^]		κ = 0.5–1.0 [µm^−1^]		κ = 1.0–1.5 [µm^−1^]		κ = 1.5–2.0 [µm^−1^]		κ = 2.0–2.5 [µm^−1^]		κ = 2.5–3.0 [µm^−1^]		Number of microtubules observed
	Breaks	Non-breaks	Breaks	Non-breaks	Breaks	Non-breaks	Breaks	Non-breaks	Breaks	Non-breaks	Breaks	Non-breaks	
20.0% compression													
1	5	4991	2	424	6	133	3	49	0	11	0	1	217
2	10	4658	3	288	1	80	1	35	1	14	0	4	206
4	14	3729	4	279	1	77	0	33	0	14	0	2	224
8	6	3697	4	306	1	93	1	32	0	9	0	1	182
12.5% compression													
4	4	3681	1	255	6	55	0	17	0	3	0	0	124
16	5	2870	1	171	2	27	0	18	0	3	0	1	112
32	0	2937	2	155	1	44	0	13	0	2	0	0	118
256	15	3319	11	243	1	73	0	12	0	4	0	0	106

## Discussion

In our previous work^16^, we determined the rate of breaking of a smoothly gliding microtubule as 0.4 ± 0.1 mm^−1^ min^−1^, and found that the breaking rate increases 250-fold from that value in an exponential fashion as the curvature increases from zero to 2.5 μm^−1^. Given that the time to complete a compression and relaxation cycle is 62 s for a compression level of 12.5% and 102 s for a compression level of 20% and the 0.5 μm segment length, we expect to a segment bent to an average curvature of 0.25 μm^−1^ to break after 10 cycles with a probability of 0.3% for 12.5% compression and 0.5% for 20% compression. These breaking probabilities should increase by a factor of 3 as the curvature is increased by 0.5 μm^−1^. The experimentally determined survival probabilities (the complement of the breaking probabilities) shown in Fig. 4 match these expectations derived from our previous analysis of microtubule breaking reasonably well.

Because the observed curvatures are below the level of 2.5 μm^−1^ up to which we previously found a consistent exponential increase in the microtubule breaking rate^16^, we assume here that the obtained two data points (12.5% and 20.0%) are in the linearly declining part of the S-N curve before a potential endurance limit is reached. Clearly, more data points at low compression levels obtained with a method allowing observation of a larger number of cycles are needed in future work, so that this assumption can be further tested. The N_50_ value of 5 × 10^6^ for 12.5% compression seems to suggest that breaking due to mechanical fatigue becomes negligible for almost all experimental situations. However, it is important to remember that the large numerical values of N_50_ simply results from the chosen segment length (0.5 μm). As Weibull discussed for the breaking of a chain consisting of several links^24^, a microtubule consisting of several segments will have a survival probability given by the product of the survival probabilities of each segment. The survival probability of a microtubule with m segments is therefore:

2 $$\:{P}_{S}\left(N,m\right)={e}^{-m{\left(N/b\right)}^{a}}$$

To determine the failure line given by the number of cycles at which 50% of the microtubules survive, one sets P_S_ equal to 0.5 and solves for N, finding that N_50_(m) = N_50_(1)/m^1/a^. For a 10 μm microtubule with 20 segments, N_50_ would be reduced by a factor 20^(1/a), that is by a factor of 2000 for a compression level of 12.5% and a factor of 40 for a compression level of 20.0%. Therefore, 50% of all microtubules would be expected to break somewhere after only 3000 (12.5%) or 30 cycles (20.0% compression). While we here treat the segments as independent from each other, the complex biomechanics of a microtubule can make the breaking probability of a segment dependent on the state of adjacent segments as described in our previous work^16^, where we found that it matters for the breaking process if a highly curved segment is at the center or the end of a bending microtubule due to a varying degree of internal stress redistribution. In the compression method used here, the sinusoidal shape of the bent microtubules yields a periodic stress distribution repeating every few micrometers. In this situation, the alternating bending prevents correlations beyond a distance of one wavelength. It therefore makes sense to consider 20–50 μm long microtubules (Fig. 1c) as consisting of independent segments and account for the variable lengths of the microtubules by dividing them into different numbers of segments. The numbers determined here for the parameters a, b and N_50_ represent averages over all assumed curvatures for a segment of length 0.5 μm, and the parameters applying to longer segments or entire microtubules can be calculated as described above. Notably, the buckling of a microtubule into a roughly sinusoidal shape is representative of several biological situations^26,27^.

The fatigue strength (Basquin) exponent B relating the stress amplitude σ_a_ to the cycle number N_50_ according to σ_a_ ~ N_50_^B^ can be estimated as -0.054±0.009, if the stress amplitude is assumed to be proportional to the maximal curvature of a buckled microtubule. Assuming a sinusoidal shape, the maximal curvature determined by the amplitude divided by the square of the wavelength and is approximately proportional to the compression level^21,23^. The fatigue strength exponent B is then given by:

3 $$\:B=\text{l}\text{o}\text{g}(20\%/12.5\%)/\text{l}\text{o}\text{g}({N}_{50}^{20\%}/{N}_{50}^{12.5\%})$$

which yields the value stated above for $\:{N}_{50}^{20\%}={10}^{3}$ and $\:{N}_{50}^{12.5\%}={5\times\:10}^{6}$. For comparison, the Basquin exponent for most metals is in the range of -0.05 to -0.12 and for some polymer nanocomposites in the range of − 0.08 to − 0.15^19,28^. This indicates that the fatigue life time increases roughly as expected if the stress on the microtubule is reduced. The two values, (5 × 10^6^; 12.5%) and (10^3^; 20.0%), determined for the S-N curve allow in principle an extrapolation to the point at which 50% of the microtubule segments break in a single cycle, which is a compression level of 30%±20%. In our experiments, even at a compression level of 40.0% not half of all – not even half of all the highly curved – segments are broken (Supplementary Fig. S1). Given the large error bar of the extrapolated compression level, it is statistically quite possible that the true required compression level for 50% breaking in one cycle exceeds 40% and is not accessible to our experimental method.

Repeated buckling of microtubules has particular relevance in the context of cardiac myocytes, where recent studies have highlighted the significant contribution of microtubules to the mechanical properties in contracting cardiomyocytes^4,29,30^. Robison et al. demonstrated that microtubules of cardiomyocytes buckle during contraction due to their attachment to the z-disks^4^. At the peak of contraction, the cardiomyocyte is shortened by 13% suggesting a comparison with our 12.5% compression data point, where a stress life of 5 million cycles, equivalent to about a month in the life of a human heart, has been determined. Curvature-induced breaking is an phenomenon observed in vivo suggesting that microtubule repair mechanisms as reviewed by Thery and Blanchoin are essential to sustain heart function^3,31^. It has to be noted though that posttranslational modifications, such as acetylation, regulate microtubule stability in cells^32^, so that the parameters measured here for paclitaxel-stabilized microtubules may be altered in specific cellular contexts. Combining the approaches for imaging microtubule self-repair mechanisms developed by Thery et al. with techniques allowing cyclical deformation such as the present method can provide additional insights in the future^17^.

Our experimental work can, of course, only describe nature and not explain it. Fatigue testing in silico pioneered by Barsegov et al. has the potential to provide explanations by making the dynamic evolution of cracks in the microtubule lattice visible^13,33^. While nanoindentation simulations model events on a timescale of milliseconds commensurate with AFM nanoindentation experiments^34^, their numerical results cannot be directly compared to our experiments conducted on a time scale of minutes and a different, but more biologically relevant mode of force application, that is cyclic bending^35,36^.

The main message from these data is that the number of microtubule breaking events for a population of microtubule segments does not increase in proportion to the number of stress cycles when the microtubules are loaded to only a subcritical level. Instead, the number of cycles required to create a given number of new breaks keeps increasing (Table 1; Fig. 4), which means that the breaking probability per cycle decreases rapidly with additional cycles. This contradicts the simple picture of mechanochemistry where each loading cycle would lead to a constant probability of bond breaking. Similar to fatigue failure at the macroscale where cracks are often initiated at randomly distributed preexisting defect sites^19^, it originates in the variability of the microtubule segments under observation, which exhibit a variety of lattice defects^31,37^. Similar to the fatigue behavior at the macroscale, small reductions in the stress level (here from 20.0% compression to 12.5% compression) have large effects on the number of stress cycles a microtubule can withstand.

## Conclusion

From a broader perspective, fatigue failure and its various aspects can be considered as a specific case of reliability theory. Gavrilov and Gavrilova highlighted the conceptual connections between biological aging and technical failure, and suggested that the characteristic time course of biological aging arises from a network of components with a preexisting random distribution of flaws, whereas technical failure follows a different time course due to the initial flawlessness of the system^38^. The microtubule is an interesting object in this context due to its variable content in mechanical defects created during its assembly^10^ and its ability to self-repair^31^. This suggests that a further exploration of microtubule fatigue failure could be fascinating.

## Experimental section/methods

### Preparation of fluorescently labeled microtubules

Tubulin was purified from fresh porcine brain using a high-concentration PIPES buffer (1 M PIPES, 20 mM EGTA, 10 mM MgCl_2_; pH adjusted to 6.8 using KOH)^39^. The purified tubulin was labeled with the ATTO 488 fluorescent dye with a labeling ratio of 1.0 as determined from the absorbance of tubulin at 280 nm and ATTO-488 fluorescent dye at 500 nm^40^. 56 µM tubulin (80% ATTO 488-labeled tubulin and 20% non-labeled tubulin) was polymerized to microtubules in the presence of 5 mM GTP, 20 mM MgCl_2_, and 25% dimethyl sulfoxide in BRB80 (80 mM PIPES, 1 mM MgCl_2_, and 1 mM EGTA, pH 6.8) at 37 °C for 30 min. Microtubules were stabilized using 50 µM paclitaxel in BRB80.

### Expression and purification of kinesin

We expressed and purified recombinant conventional kinesin-1 construct consisting of human kinesin (residues 1-573) as described previously with partial modification^41,42^.

### Preparation of the flow cell

We used PDMS film with a thickness of 0.05 mm as an elastic substrate to prepare the flow cell. The relaxation and stretch cycles were performed using a previously developed micro-stretcher consisting of a base plate containing a computer-controlled stretcher/compressor and a cover plate^20,21^. First, the PDMS film with approximate dimension 4.0 × 5.0 × 0.05 mm^3^ (L×W×T) was fixed horizontally on the stretcher. Then the film was elongated 100% by applying tensile force with the computer-controlled stretcher. This pre-stretched PDMS film was masked with two 18 × 18 cover glasses, leaving a narrow channel for use as a flow cell. The channel was exposed to etching with a plasma etcher (6–8 Pa, 8 mA, 4 min) to increase the hydrophilicity prior to each experiment. The flow cell was then incubated for 5 min with 10 µL of 50 nM kinesin in buffer A (80 mM PIPES, 40 mM NaCl, 1 mM EGTA, 1 mM MgCl_2_, 0.65 mM DTT, 1.3 mg/mL casein, 13 mM paclitaxel; pH 6.8) and washed with buffer A. Next, 20 µL of paclitaxel-stabilized, ATTO-488-labeled microtubule solution (280 nM tubulin) was introduced by flowing the solution from one end and holding filter paper to the other end of the channel which ensured that the microtubules are aligned parallel to the stress axis and incubated for 5 min, followed by washing with buffer A. This results in microtubules bound to the PDMS substrate via interaction with kinesin with a parallel alignment relative to the stretch axis. The micro-stretcher chamber was then sealed, and humid nitrogen gas was continuously purged through until the observation finished. The concentration of the kinesin was the same in all experiments.

### Repetitive cycles of relaxation-stretching of PDMS

The pre-stretched PDMS was relaxed by a chosen strain (10.0, 12.5, 20.0, 40.0% of the pre-stretched length) to compress the microtubule attachment points and sinusoidally buckle the microtubules. The choice of the strain level was limited by our observation system, which allows up to 40.0% compression of the PDMS from its pre-stretched length. Repetitive cycles of relaxation and stretching were carried out until breakage of the microtubules was observed. The number of applied repetitive cycles were chosen as power of 2, that is, 2^0^, 2^1^, 2^2^, … 2^8^. During each set of repetitive cycles PDMS was relaxed and then returned to the initial stretch, always operating with a strain rate of 0.004 s^−1^ and a 1 s gap between movements. Microtubules were imaged after each motion (compression or stretching) was completed. Breaking events were identified by visually inspecting the fluorescence signal along each microtubule and identifying inhomogeneities. After the prescribed number of compression and stretching cycles was completed, the substrate was stretched by an additional 2.5% and imaged. This revealed additional breaks (22 for 12.5% compression and 140 for 20.0% compression) which were not included in the analysis, because it was not clear when these breaks occurred and it was not assured that the breaks were a result of compression only and not the final 2.5% stretching.

### Generation of survival curves and estimation of N_50_ values

Survival curves were constructed using the Kaplan Meier estimator; the survival probability, *S(j)*, after a set of *j* compression cycles can be written down as the product.

4 $$\:S\left(j\right)={\prod\:}_{i\le\:j}(1-\frac{{d}_{i}}{{r}_{i}})$$

where *d*_*i*_ the number of breaking events during *i* cycles and *r*_*i*_ denotes the number of microtubules at risk during i cycles. Pointwise standard errors of survival probabilities were estimated using Greenwood’s formula:

5 $$\:{\sigma\:}^{2}\left(j\right)=S{\left(j\right)}^{2}{\sum\:}_{i\le\:j}\left(\frac{{d}_{i}}{{r}_{i}({r}_{i}-{d}_{i})}\right)$$

where *σ(j)* is the pointwise standard error for the estimate of the survival probability *S(j)*. The number of cycles required to break 50% of the microtubules N_50_ was estimated by fitting the following model:

6 $$\:{\text{log}}_{10}S\left(j\right)=\frac{{\text{log}}_{10}\left(0.5\right)-b}{\gamma\:}\cdot\:{\text{log}}_{10}\left(j\right)+b$$

where the equation is parametrized such that b is an intercept term and γ corresponds to the $\:{\text{log}}_{10}{N}_{50}$. The above equation is fit using least squares regression with the σ(j) derived from Eq. (5) used as weights. Fitting is conducted using the *nls* function from the R ‘stats’ package. Standard errors from the fit for *γ* are reported as error bars.

## Electronic supplementary material

Below is the link to the electronic supplementary material.

Supplementary Material 1

## Data Availability

The original experimental recording used as the basis of this study are available at: 10.5061/dryad.6hdr7sr3v.
