# Supplementary material for: Mechanical fatigue in microtubules
Source: Sci Rep. 2024 Nov 1;14:26336. doi: 10.1038/s41598-024-76409-7 (PMC11530518; doi:10.1038/s41598-024-76409-7)
Supplement: Supplementary file 1 — Supplementary Material 1 [file 41598_2024_76409_MOESM1_ESM.pdf]

SUPPLEMENTARY INFORMATION

Mechanical Fatigue in Microtubules

Syeda Rubaiya Nasrin[1], Neda M. Bassir Kazeruni[2], Juan B. Rodriguez III[2], Stanislav Tsitkov[2,3], Akira Kakugo[1], Henry Hess*[2]*

1. Division of Physics and Astronomy, Graduate School of Science, Kyoto University, Kitashirakawa-Oiwake-Cho, Sakyo-ku, Kyoto, Japan 606-8502

2. Department of Biomedical Engineering, Columbia University, 1210 Amsterdam Avenue, New York, NY 10027, USA

3. Department of Biological Engineering, Massachusetts Institute of Technology, Cambridge, MA, USA

*Corresponding Authors: kakugo.akira.8n@kyoto-u.ac.jp, hh2374@columbia.edu

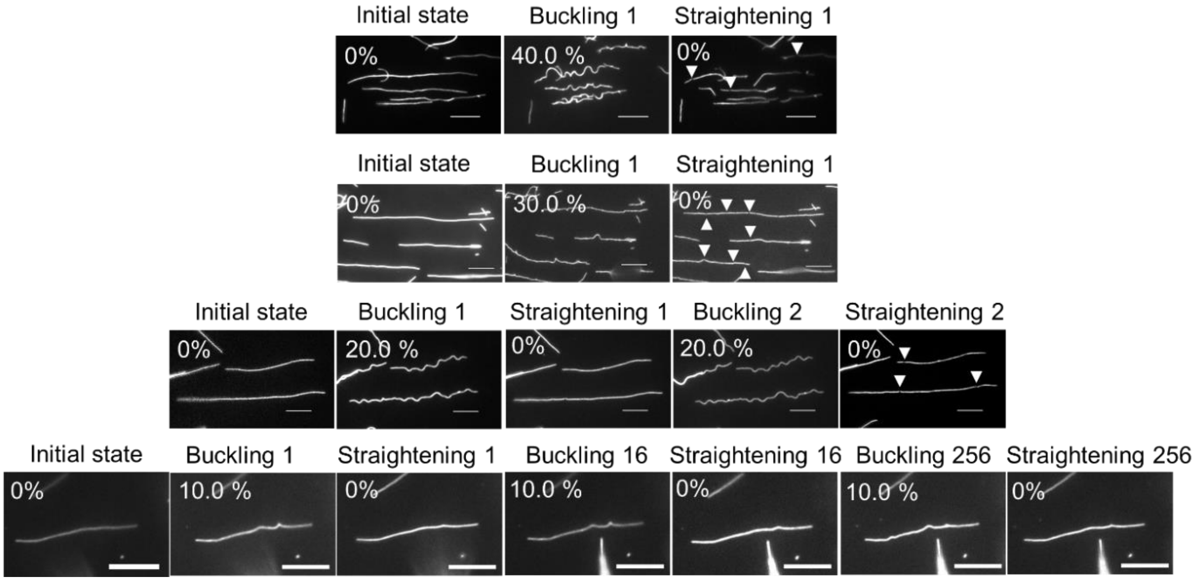

Supplementary Figure 1. Images of representative microtubules at different compression levels. Representative fluorescence images showing microtubule conditions after buckling-straightening cycles at different compression levels 0-40% (top row), 0-30% (2nd row from top), 0-20% (2nd row from bottom), and 0-10% (bottom row). White arrow heads indicate the positions of breakage or damage of the microtubules after certain numbers of cycles. Scale bar: 10 μm.

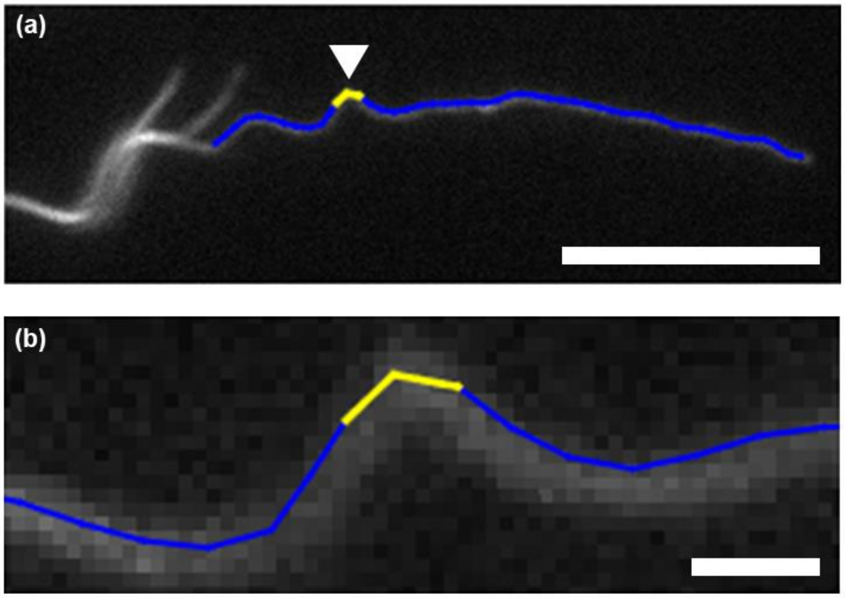

Supplementary Figure 2. Image of microtubule under 30% compression broken down into segments used for curvature calculation. White arrow heads indicate the positions of breakage. The two segments used to calculate the curvature of the breaking event are shown in yellow, while non-broken segments are in blue. Scale bar: 10 μm (a) and 1 μm (b).

Supplementary Videos (Captions)

Supplementary videos are available at: https://doi.org/10.5061/dryad.6hdr7sr3v

Supplementary Videos 1-5 | 12.5% Compression Assays.

Videos of the compression assays of fluorescently labeled microtubules undergoing 12.5% compression on flexible PDMS surface coated with kinesin-1. Each video contains 13 frames, two for each measured compression cycle (1, 4, 16, 32, 64, and 256), alternating between the compressed and subsequent relaxed stage of the cycle. The final frame of each video is a stretch of 2.5% after 256 compression cycles that is used to identify breaking events. Field of View: 277.33 µm x 234.00 µm.

Supplementary Videos 6-15 | 20% Compression Assays.

Videos of the compression assays of fluorescently labeled microtubules undergoing 20% compression on flexible PDMS surface coated with kinesin-1. Each video contains 9 frames, two for each measured compression cycle (1, 2, 4, and 8), alternating between the compressed and subsequent relaxed stage of the cycle. The final frame of each video is a stretch of 2.5% after 8 compression cycles that is used to identify breaking events. Field of View: 277.33 µm x 234.00 µm.
